# ‘In my experience …’: The use of the word *experience* in peer online forums for mental health

**DOI:** 10.1177/20552076251385593

**Published:** 2025-11-13

**Authors:** Anna Lindroos Cermakova, Elena Semino, Karin Tusting, Neil Caton, Matthew Coole, Zoe Glossop, Steven Jones, Christopher Lodge, Paul Marshall, Tamara Rakic, Paul Rayson, Heather Robinson, John Vidler, Fiona Lobban

**Affiliations:** 1 Division of Health Research, 4396 Lancaster University, Lancaster, UK; 2 Linguistics and English Language Department, 4396 Lancaster University, Lancaster, UK; 3 41865 UK Centre for Ecology and Hydrology, Wallingford, UK; 4 Spectrum Centre for Mental Health Research, 4396 Lancaster University, Lancaster, UK; 5 School of Computing and Communications, 4396 Lancaster University, Lancaster, UK; 6 Department of Psychology, 4396 Lancaster University, Lancaster, UK

**Keywords:** Mental health forums, lived experience, lived expertise, corpus linguistics, online peer support

## Abstract

**Objective:**

Peer support online forums potentially offer accessible and inexpensive access to information and support through shared lived experience, including in relation to mental health. However, the impacts of participating in online communities are not fully understood. The present study takes a linguistic perspective to investigating how references to personal lived experience are (1) used, that is, how forum contributors present their *experience* and (2) responded to, that is, how forum contributors react to *experience* of others.

**Methods:**

The study employs the methods of corpus-based discourse analysis using data from two mental health forums. The study design and results have been conducted in consultation with a PPI group.

**Results:**

When sharing what they call their *experience*, forum contributors typically give advice and/or provide information for the benefit of others. The most frequent information type is ‘information about treatment and medication’, while the most frequent advice type is ‘advice to seek help’. When contributors respond to what they call others’ *experience*, they typically express gratitude and reciprocally share their own experience. In some cases, they also explicitly articulate the impact of reading others’ experience, for example, by saying that they feel *less alone*.

**Conclusion:**

While we found some instances of negative judgements about health professionals, we did not find any clearcut instances of mis/disinformation or potentially harmful advice. Overall, the analysis supports the view that sharing lived experience in peer online mental health forums can be beneficial.

## Introduction

Mental health problems affect approximately one in six people in the United Kingdom.^1^ The World Health Organisation recently identified insufficient supply of mental health services as a primary barrier to mental healthcare worldwide.^2^ Against this backdrop, peer support online forums potentially offer an easily accessible and inexpensive alternative and/or additional type of support to professional support and face-to-face peer support groups. Many forums are available 24/7 and thus have the potential to deliver support at scale.^3^ Indeed, there is evidence of a growing use of such forums.^4,5^

Marshall et al.^3^ define ‘mental health peer support forums’ as ‘online, primarily asynchronous text-based discussion platforms’ that encompass ‘any forum primarily intended to support people experiencing psychological distress, including those with specific mental health difficulties, experiencing isolation, substance misuse or addiction, or caregiving for someone with a mental health difficulty’. Such online forums are perceived by many as providing convenient access to information and advice in a non-judgmental environment, where what is referred to as ‘lived experience’ can be shared for the benefit of others and oneself.^6^ As such, they offer emotional support and may support patient autonomy by complementing the information provided by clinicians.^7,8^

Smailhodzic et al.^9^ conducted a systematic literature review of the effects of social media use by patients for health-related reasons. They identify several types of use, such as seeking emotional support, information, and network support. These, in turn, may potentially lead to patients’ improved self-management and control as well as enhanced psychological and subjective well-being, but also diminished well-being and loss of privacy. The research also highlights that social media use can affect the patient–healthcare professional relationships and dynamics.

The role of informational and emotional support in online health communities has been analysed extensively,^10^ showing that seeking information about a condition and treatment options may make patients better prepared for consultations with a medical professional.^11,12^ By sharing information and experience with peers who are well equipped to sympathise with their circumstances, patients may feel more informed and less lonely.^13,14^ Even passive participation, that is, not actively contributing and only reading others’ stories, may reduce anxiety.^15^ Feeling more informed about their condition and learning about others’ coping strategies may also improve patients’ perceived control and ability to manage their condition.^15^

The importance of the supportive role of social networks has been underscored in several research studies. Gruzd and Haythornthwaite^16^ compared offline and online relationships and concluded that, generally, social bonds maintained online can be as supportive as face-to-face interactions. The networks can convey a sense of belonging, help combat loneliness and compensate for the lack of face-to-face social interactions. This applies particularly to networks of participants with shared attributes,^17,18^ as in the case of patients experiencing mental health challenges, who are more likely to share their health concerns with peers experiencing similar circumstances.^19^

However, in spite of the increasing popularity of mental health forums, the impacts of participating in online mental health forums, including by sharing and receiving lived experience, are not fully understood, with concerns being raised particularly around their safety and effectiveness. In general, research suggests that internet users are not sufficiently equipped to assess the quality of information received through these channels.^20^ Indeed, previous research has identified both positive and negative impacts.^9^ On the one hand, online forums have also been shown reduce mental health-related issues such as social isolation, depression, and suicidal thoughts.^21–23^ On the other hand, they can aggravate other related issues such as suicidal ideation, negative body image, and disordered eating patterns.^24,25^ In order to make decisions about the use of forums in mental health support, therefore, there is a need to better understand how these impacts are generated, across different forums, and for different people.

Online participation in general is increasing and research topics reflect this change – recent research studies focus, for example, on technological affordances of online communities and how they can co-create value and provide access to support^26^ or on specific at-risk subgroups, such as migrant communities.^27^ Other recent studies have focused on advice-seeking, advice-giving, and advice-evaluation to determine which advice is deemed by the participants as helpful^28–32^; for example, Reuger et al.^28^ show that speedy advice received from others, who have similar predominant interests, is valued most. Other studies^33^ focus on evaluating the quality of information received in online forums, acknowledging the issues arising from the potential misinformation while also recognising that well-designed online communities ‘can be safeguards of high-quality information, but new models will need to be developed that will allow a higher level of integration of health professional moderators and the implementation of mechanisms to attenuate the sharing of low-quality information among users’ (p. 16).

Many studies have focused on the language used in the forums. For example, Sillence^10^ highlights the role of narrative, as narratives or personal stories are one way in which people convey information about their health and well-being in the internet communities. Narrative is a form of social practice which captures habituality and regularity in discourse^34^ and affords the opportunity to study how people make sense of their experiences.^35^ Some studies focusing on narrative tend to treat the stories as an authentic window into a teller's experience,^34,35^ while potentially insufficiently considering that ‘language use can – and usually is – shaped by motivations other than simply being “transparent” or “authentic”’ (p.-34). Other research on narrative focuses, for example, on complexities of lay interpretation of expert discourse.^36^

In addition to narrative, other examples of language-based approaches include LIWC (‘linguistic inquiry and word count’) methodology and some NLP (‘natural language processing’) techniques^37^ (for extensive critical discussion of LIWC in the context of forum analysis, see Hunt and Brookes.^38^ While NLP methods focus on the use of words in isolation, in this article we employ the methods of corpus-based discourse analysis, which involve the construction of large digitally searchable textual datasets (or ‘corpora’) and the combination of both quantitative and qualitative methods to the study of repeated linguistic patterns in their context of use. These methods have proved useful in several areas of health research, including, for example, cancer^39–42^ and end of life care.^43,44^ While one's experience sharing may linguistically take many forms, the present study focuses only on the use of the word *experience*. In selected peer online mental health forums, we systematically investigate how this word is used by forum contributors in reference to their own or others’ lives. Of course, this precise and narrow focus on one word offers only a snapshot of how forum contributors talk about their experiences; however, we are interested specifically in what forum contributors do when they explicitly signal that they are relating or responding to their own or others’ *experience*.

In addition, our study shows the potential of an interdisciplinary approach. Corpora consisting of forum posts, that is, non-elicited, spontaneously produced text, present the analyst with rather ‘messy’ data but are a useful complement to other approaches that investigate elicited responses, for example, surveys and interviews. This study is situated within the context of the Improving Peer Online Forums (iPOF) project, (https://www.lancaster.ac.uk/ipof/) which uses a transdisciplinary approach to understand the mechanisms underpinning the positive and negative impacts of online mental health communities, with the aim of co-designing good practice guidance and tools, with relevant stakeholders.

The objective of this article, therefore, is to answer the following research questions:

1. How do forum contributors use what they refer to as their own *experience* in their posts?
2. How do forum contributors react in their posts to what they describe as *experience* shared by others?

## Methods

### Data: Forums used in this study

In this article, we analyse two corpora consisting of posts from two online peer support forums dedicated to mental health, which we will refer to as Starling and Magpie. Both forums have wide user communities but differ in other respects. Starling is a user-led forum on a commercial site open to everyone, with a broad thematic focus on mental health. It has non-professional volunteer moderators, who are active users of the community with their own lived experience. Magpie consists of a platform of multiple sub-forums for health-related discussions, which are typically created on behalf of predominantly voluntary, community and faith sector (VCFS) organisations. Our dataset includes a subset of four Magpie sub-forums; each focused on a specific mental health topic or condition. Magpie forums involve moderators who are trained by the organisations that created them. A more detailed description of each forum can be found at the iPOF project website (https://www.lancaster.ac.uk/health-and-medicine/research/spectrum/research/ipof/case-summaries/). The data includes posts made between 2016 and 2023, both from the forum users and their moderators; however, due to ethical concerns, we have, in this study, anonymised the data so that we cannot link the posts back to the individuals that posted them or distinguish between users and moderators. The total number of posts in the dataset is 146,388, which amounts to 14,621,374 words (Table 1).

**Table 1.** Word and post counts in the datasets.

	Number of words	Number of posts
Starling	4,820,253 (33%)	46,868 (32%)
Magpie	9,801,121 (67%)	99,520 (68%)
Total	14,621,374 (100%)	146,388 (100%)

### Ethical considerations

Ethical approval for the iPOF study was granted by the UK National Health Service (NHS) Solihull Research Ethics Committee (IRAS 314029). The study is sponsored by Lancaster University and hosted by Berkshire NHS Foundation Trust in the United Kingdom.

In analysing forum data, that is, posts by the forum users, there are numerous important ethical issues.^45^ We have carefully considered the potential costs and benefits of carrying out this research, and worked closely with multiple stakeholders, legal and governance experts, and forum hosts, moderators and users to develop a comprehensive ethical framework which is available on the iPOF study website. (https://www.lancaster.ac.uk/health-and-medicine/research/spectrum/research/ipof/ethics-framework/) The wider objective of this article, to understand one of the ways in which lived experience is explicitly used on online forums, supports the overall goal of designing forums that better support their users’ needs. In doing so, we recognise the importance of ensuring forums remain a safe space to share information and that maintaining the anonymity of users is fundamental to this sense of safety.

The Starling forum is a publicly open forum with no expectation of privacy. In collaboration with the forum moderators, we posted about the study onto the forum, with a designated email inviting questions and debate. We gave users the option to email if they wanted their posts removed from the dataset. Users of Magpie are able to freely give consent at sign up (i.e. they can still use the forum without consenting for their data to be used). Consequently, in this study, we only used posts made by consenting users of Magpie.

All data from both forums was anonymised before being analysed, including removal of usernames, and place or person's names. De-identified data has been stored in a Secure Research Environment with restricted access and will not be shared as part of the Open Science Framework. Our de-identifying efforts further include pseudonymisation of the forum names (using bird names, i.e., Starling and Magpie in this study) and the modification of all examples quoted so that they cannot be linked directly back to the forum. Moderators have not been identified within the dataset as this increases the likelihood of identification; though their role is likely to be significant.^46^

### The study keyword: The word ‘experience’

Our study investigates experience sharing on mental health online forums by analysing posts that use the word *experience* itself. While lived experience can be linguistically expressed in a variety of other ways, our approach enables us to focus on cases where the authors of the posts themselves present what they are reporting or responding to as their own or others’ experience.

We initially considered including the term *expertise* alongside *experience* to collect relevant posts from our datasets, as ‘lived experience’ and ‘lived expertise’ are both used to refer to the knowledge and wisdom that are recognised as a valuable resource in mental health services, as well as in policy and clinical guidelines development (e.g. NICE guidelines in the United Kingdom). Newton et al.^47^ state that ‘the concept of lived experience recognises the knowledge held by individuals with first-hand experience living with a specific identity or living through a particular event’, which ‘translates’ into lived expertise. However, as Newton and colleagues also point out, these two notions are often conflated in the literature.^30,48^

But, from a linguistic point of view, the terms *experience* and *expertise* are only marginally synonymous. More importantly, they differ markedly in frequency of use, both in the English language generally and in our datasets. According to the Oxford English Dictionary (OED),^49^ the frequency of the word *experience* in modern written English is about 200 occurrences per million words, while for *expertise* it is about 20 occurrences per million words. This tenfold difference suggests that the word *expertise* is much rarer and more specialised than *experience*.

In addition, in our data, *experience* has 687.6 occurrences per million words (10,054 occurrences in total), while *expertise* has only 7.9 occurrences per million words (115 occurrences in total). This suggests that the word *experience* is highly relevant to our dataset, while *expertise* is not. Our study is therefore focused on uses of the word *experience* only.

Corpus linguistic analysis typically starts from a keyword in context, *experience* in this case, and gradually expands the analysis based on quantitative indicators, for example, frequencies of co-occurrences of words, to identify typical relevant patterns. After narrowing the analysis in this way, a qualitative analysis is performed. We therefore began by loading the data onto a corpus tool^50^ and ran a collocate profile for *experience*. A collocate profile shows what words are frequently used in close proximity to the search word (in our case, *experience*) to a statistically significant extent. This, in turn, allows the analyst to focus on typical and frequent discourse patterns that may reveal habitual behaviour. The top 15 collocates of *experience* in the data, based on likelihood statistics, are provided in Table 2 (NB: ‘pp’ in line 11 is an acronym for a mental health condition).

**Table 2.** Top 15 collocates of the word *experience*, calculated based on Likelihood statistical measure calculated within a context of five words to the left and right of *experience*.

Rank	Collocate	Frequency	Range	Likelihood	Effect
1	personal	476	13	1900.650	4.235
2	my	2666	18	1845.456	1.380
3	lived	258	11	1317.009	5.065
4	with	1943	18	1312.748	1.364
5	had	1101	16	1280.379	1.886
6	from	1120	15	1251.477	1.841
7	traumatic	229	12	1015.368	4.568
8	share	393	14	995.485	3.096
9	of	2585	18	922.052	0.947
10	similar	296	13	899.595	3.501
11	pp	413	4	832.012	2.664
12	sharing	288	15	766.779	3.201
13	own	393	16	573.169	2.171
14	have	1751	17	560.347	0.895
15	your	1443	18	486.177	0.922

The list of the top collocates (Table 2) includes, in addition to ‘content’ words like *personal*, *lived*, *traumatic*, *similar* and *sharing*, two possessive expressions: *my* (line 2 in the table, with 2666 co-occurrences) and *your* (line 15 in the table, with 1443 co-occurrences). As the forum interactions can essentially be viewed as conversations among the participants, these combinations are particularly relevant to our concern for how references to personal experience are used – *my experience* – and responded to – *your experience*.

We therefore further extracted the collocates of the phrases *my experience* and *your experience*. The top three collocates of *my experience* are the words *in*, *share* and *from*. The top three collocates of *your experience* are *sharing*, *sorry* and *thank*. An initial exploration of these collocational pairs led to the decision to focus on the following three patterns as most relevant to our research questions: *from my [X] experience*, *in my [X] experience*, for the purposes of RQ1; and *sharing [X] your experience(s)* for the purposes of RQ2 (NB: ‘X’ indicates there may be one or more intervening words; our searches included both the singular and plural forms of the noun *experience*).

All instances of the three phrases were extracted from the data, resulting in:

- 307 instances of *from my [X] experience(s)* (hereafter *from my experience*);
- 362 instances of *in my [X] experience(s)* (hereafter *in my experience*);
- 272 instances of *sharing [X] your experience(s)* (hereafter *sharing your experience*)

### Coding ‘experience’

#### In/from my experience

In combination, the phrases *in my experience* and *from my experience* have a total of 669 instances, of which 32% were from Starling and 68% from Magpie, which reflect the data proportions in our corpus. In order to answer RQ1, we designed a coding scheme that aimed to capture what posters do in their posts when they use one of the two phrases. The coding scheme was based on a random sample of 30 instances and was developed through an iterative bottom-up process involving three coders with expertise in Linguistics. After several rounds of discussion and coding adjustments to reflect the data and the required level of granularity, a mental health service user researcher within the team was consulted to help refine the final version.

The coding scheme captures the two main types of linguistic behaviours that were found to occur in close proximity to the phrases *in/from my experience*, namely, providing advice and providing information.

We operationalised ‘advice’ as the use of verbs in the imperative mode (e.g. *try X medication*) or of lexical items such as *should*, *advice* or *suggest,* in relation to a course of action that is presented as beneficial to the addressee. For example, in (1) below, the poster employs the phrase *I really suggest* in order to recommend to the addressee two further online communities that, based on their *past experience*, may prove helpful:

(1) but **I really suggest** you check out [WEBSITE NAME] to see that you are not the only one going through this and [WEBSITE NAME] which is a bigger group and a highly supportive community **from my past experience**
  We operationalised ‘information’ as statements concerning processes, events or facts that are based on the person's experience but are presented as potentially of more general applicability. In (2) below, the poster draws from their *personal experience* to make general statements about medication.
(2) Actually, medication can be useful in addressing various symptoms which arise from [NAME OF CONDITION] when it is not treated. This is something I’m taking **from my personal experience**. To reiterate, this is something that should be put in place by the professional.

Based on our familiarity with the data, proximity was operationalised as two sentences on either side of the sentence in which the phrase occurs, to ensure that instances of advice/information were close enough in the post to the reference to the person's experience for us to reasonably assume that they were linked.

Each instance of advice or information was further coded for the topic(s) of the specific case of advice/information. The topic codes with examples are provided in Tables 3 and 4. The main topic codes (four for advice and five for information) capture the most frequent patterns in the data. The ‘other’ codes were applied to cases where the topic was not part of a larger pattern and/or not directly relevant to mental health. Each example could in principle be coded as including both advice and information, and receive up to three advice and/or information codes.

**Table 3.** Advice topics with examples.

Advice topic code	Example
Advice to seek help	I’m talking **from my own experience** […] don't give up on getting the help, just keep trying […] you’ll get there in the end.
Advice about health professionals	I’d say you should go to your doctor and talk to them about how you are feeling […] **from my experience**, when circumstances change it can really be a trigger […] you can also contact […] or […] for advice confidentially
Advice about treatment and medication	**In my experience** with […] various medications, if you take the time to adjust to it, it will pay off […] initially I had some side effects for a few days […]
Advice about non-medical management of MH* condition	Giving her something might help […] **from my own experience** and my family's I discovered […] that saying things is easy […] but as well, small actions can change a lot.

*MH = mental health.

**Table 4.** Information topics with examples.

Information topic code	Example
Information about diagnostic process	**In my experience** a couple of things will follow. Your doctor will first talk you through a questionnaire. Then, they will order some tests.
Information about condition	I felt as if [CONDITION] was altering my identity […] So i**n my personal experience** it could indeed be the case that […] is having an effect on aspects of your character
Information about health professionals	The answer is yes - but the patient needs to be very determined - and it needs help from professionals like – counsellors and dieticians - […] - you’ll have to ask your doctor for a referral to a community service - [it] takes rather a long time - **from my experience** it can be more than six months. [HELPLINE] support people and their advice is really helpful, you could get in touch with them.
Information about treatment and medication	[…] if you’ve taken it before […] and your reaction was ok […] they are often a bit wary, **from my experience** at least, about re-starting the meds under a generalist doctor
Information about non-medical management of MH* condition	Maintaining contact with your [FAMILY MEMBER]? A good diet, staying hydrated and keeping active? I know these little things might seem rather obvious but **from my** own **experience** when my life was at its worst crisis point, in desperation, […], I was kept safe by just doing the small “sensible” things, marking them off, and writing out a simple daily plan.

*MH = mental health.

#### Sharing your experience

Out of the 272 instances of *sharing your experience*, nine cases involved invitations to share experiences, or general statements about the usefulness of doing so. As we were interested in how posters react to what they describe as someone else sharing their experience (cf. RQ2), these nine cases were excluded from the analysis. This resulted in a set of 263 occurrences, of which 83% were from Magpie and 17% from Starling. All of these examples were found to additionally include some form of expression of appreciation for sharing in the forum, usually realised via *thank you* (249 cases).

To answer RQ2, a coding scheme was developed to capture the main types of reaction to someone else sharing their experience. The process for developing the coding scheme was the same as for the coding of *in/from my experience* in the previous section. This resulted in nine reaction codes, divided into two broad groups: ‘interactional’ and ‘impact’. The codes in the ‘interactional’ group capture different ways in which the poster may respond to the person who shared the experience. They include: ‘expressing sympathy/concern’, ‘expressing empathy due to similar experiences’, ‘sharing more personal details about oneself’, ‘asking for more details about the person's experience’, ‘giving advice’ and ‘offering further support’ (see Table 5 for examples).

**Table 5.** Interactional codes with examples.

Type of interactional reaction	Example
Expressing sympathy/concern (‘feeling for’)	Hello X Thank you for **sharing your experience**. I’m sorry to hear you have had so much struggle and suffering ….
Expressing empathy due to similar experiences (‘feeling with’)	Hi X Thanks so much for **sharing your experiences** of […]. I recognised them easily, my own experiences were very similar
Sharing more personal details about oneself	It's a really hard topic to address, because everyone is different […] At my initial diagnosis they told me […] the pain I experience every day is chronic and debilitating […] and that's the reason I said yes to the surgery. Your words are kind, I thank you for that & for **sharing your experience** with me.
Asking for more details about the person's experience	Hi thank you for **sharing your experience**. I discussed it with my husband and […] Are you and your partner still a couple, have things improved?
Giving advice	I can appreciate why you are confused […] I wanted to point you in the direction of the […] website and Insider Guides, unless you’ve seen them already […] also can also provide a second opinion
Offering further support	If there are any particular questions you have […] if I can give you any information I would be happy to do so. Do get in contact by direct message or email X

The codes in the ‘impact’ group capture the effect that what is described as the other person's *experience* has had or may have in the future on the current poster. They include: ‘feeling better/less alone/more supported’, ‘hoping to feel better’ and ‘intending to take action’ (see Table 6 for examples). ‘Feeling better/less alone/more supported’ captures a self-reported change in emotional state that is presented as having occurred as a result of reading the experience shared by the other person. ‘Hoping to feel better’ captures delayed impact – the person says that, based on the other person's experience, there is hope for them, or their loved ones, to feel better in the future. ‘Intending to take action’ refers to any next steps the poster says they intend to take based on what they describe as the *experience* shared by the other person.

**Table 6.** Impact codes with examples.

Type of impact reaction	Example
Feeling better/less alone/more supported	Thank you for **sharing your experiences** too, I think you’re right […] I probably wouldn't have lived to tell the tale […] for all of you and the support you offer, which is incredible. Thank you always for keeping both of us safe.
Hoping to feel better (delayed impact)	I’m taking this for [CONDITION], thank you for **sharing your experience** with it, I hope it is effective for me as well.
Intending to take action	Thanks for describing such a detailed account and **sharing your experience**, I really appreciate it. […] I will absolutely have to talk to my doctor about this.

Based on our familiarity with the data, the different types of reactions could occur anywhere in a post and still be reasonably assumed to be linked to reading the other person's experience. Therefore, the textual scope for identifying instances of reactions was the whole post.

## Results

### In/from my experience

The analysis of the uses of *in/from my experience* (see Table 7) shows that information codes are more frequent overall (present in 61% of posts) than advice codes (12%). In about a quarter of posts that include *in/from my experience* (26%) both codes are present. Only a small minority of posts contain neither advice nor information (1%).

**Table 7.** Overview of distribution *of in/from my* experience codes.

Code	Frequency (percentage of posts and number of posts)
Advice only	12% (83 posts)
Information only	61% (408 posts)
Advice and information	26% (173 posts)
No advice/information	1% (6 posts)

**Figure 1.**
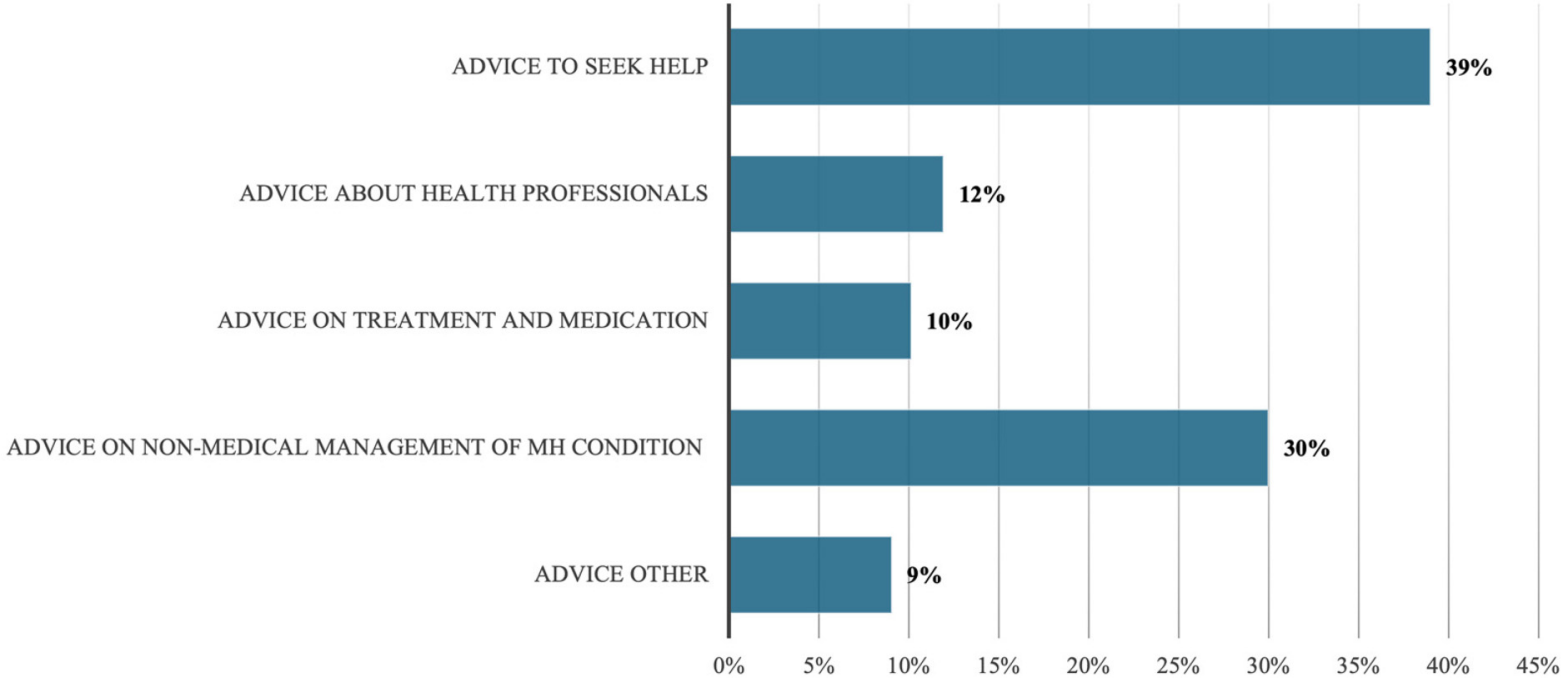
Proportion of advice topic codes out of all advice codes.

**Figure 2.**
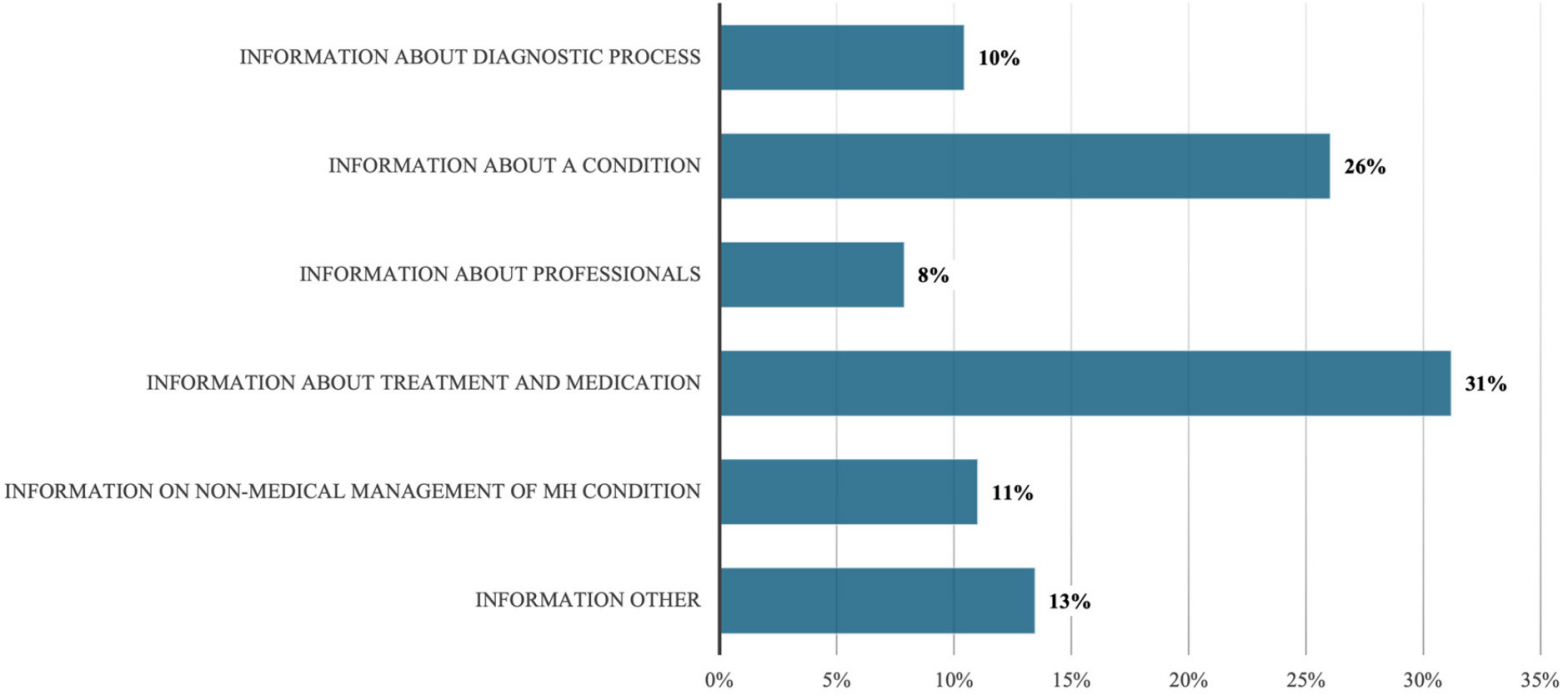
Proportion of information topic codes out of all information codes.

Figures 1 and 2 provide an overview of the different topic codes for information and advice, respectively. Among the advice codes (Figure 1), the most frequent are ‘Advice to seek help’ (39% of all advice codes) and ‘Advice on non-medical management of MH condition’ (30%). Among the information codes (Figure 2), the most frequent topic codes are ‘information about treatment and medication’ (31% of all information codes) and ‘information about a condition’ (26%).

Our findings with regard to the frequencies of different types of advice and information reveal several common patterns in our data whereby posters use their own experience, referred to as *experience*, to advise others to engage with (offline) mental health services and/or seek additional sources of help. Examples (3) and (4) below exemplify, respectively, the codes ‘advice to seek help’, and ‘advice on non-medical management of MH condition’:

(3) Do try a therapist. It's necessary. **From my experience** they are a great help.
(4) It's good to try and have 8 hours sleep too. A sleep routine has so much more to it […], how to react if you wake up in the small hours. But **from my experience** it is worth it.
  In addition, both advice and information are provided about how to engage with mental health services, and what to expect. Examples (5) and (6) exemplify, respectively, codes ‘advice about health professionals’ and ‘information about health professionals’. These two examples also point to two recurring themes: in example (5) the poster points to possible communication difficulties and how to deal with them, while in example (6), the poster highlights the long waiting times for NHS services.
(5) If you discover that what they [psychologists and counsellors] are meaning doesn't make sense to you, please ask […] being honest with them is much better […] I know **from my** own **experience**…
(6) You can find people to help out there, but **from my experience** the choice is either the NHS with a wait or paying for treatment yourself.

Among the information codes, perhaps as one might expect, the most frequent codes are ‘information about treatment and medication’ (example 7) and ‘information about a condition’ (example 8), in which posters share details related to their condition and how it has been dealt with.

(7) I’ve had three separate times taking […] I am just restarting them for the fourth time, now into week 2… I know **from my experience** that at around the end of October I will start to feel an improvement. Stick with it for a few more weeks and then if you still don't feel any different, talk to the doctor, there are lots of different possible types out there that you can try.
(8) **In my experience** autoimmune conditions can be caused, or at least triggered, by mental health problems and stress.

Somewhat less frequent are the topics that were coded as ‘information about diagnostic process’ (example 9) and ‘information about non-medical management of MH condition (example 10).

(9) Even if you try something serious, **from my** own **experience** you’ll have some blood tests, you’ll be kept waiting and then …
(10) **From my experience** it takes long time to switch off […]. I find that if I ensure I have planned things to do […] like crafting, going out, seeing people, etc it helps me to think about other things – best of luck with your recovery.

Some posters make negative evaluations of mental health services especially in terms of long waiting times (see example (6) above) and sometimes advise others to opt for private providers as opposed to state-provided healthcare (e.g. ‘I would recommend […] going private and not bothering with the NHS at all’). However, our analysis of the use of *in/from my experience* overwhelmingly shows a focus on how others can best navigate and make the most of healthcare services, professional expertise and mainstream treatments and medication. With regard to the latter, several posters provide advice or information to the effect that it is worth persisting with medication despite side effects or even if benefits are not immediately obvious. For instance, example (7) above highlights that benefits may take some time to be felt.

For both advice and information, a substantial proportion of codes focused on non-clinical help. These tend to be concerned with healthy lifestyles (see examples 4 and 10 above) but also very often with relationship management with family, friends and at work, as in example (11):

(11) That's something I’m going through right now and it isn't much fun. **From my experience** right now I’d say having people around me who understand and care is really the thing that's helping me with my […] anxiety.

### Sharing your experience

Having looked at how experience is offered in the previous section, in this section we examine how posters respond to what they describe as *experience* shared by others. Posts containing the phrase *sharing your experience* do not necessarily occur in response to a post that includes *my experience*. Rather, these posts show how the forum participants react to something that they see as the addressee's experience, whether or not the other person explicitly describes it as such. Table 8 below shows that interactional codes, for example, expressing empathy or sharing personal information, are, perhaps expectedly, much more frequent: 77% of the posts that include *sharing your experience* have one or more interactional codes and another 14% have both interactional and impact codes (i.e. explicit expressions describing impact, e.g., ‘feeling less alone’). Posts that have only impact code(s) are relatively rare (3%).

**Table 8.** Distribution of impact and interactional codes across the dataset.

Code	Frequency (percentage of posts and number of posts that include sharing your experience)
Impact code(s) only	3% (9 posts)
Interactional code(s) only	77% (202 posts)
Both impact and interactional codes	14% (38 posts)
No impact/interactional codes	5% (14 posts)

**Figure 3.**
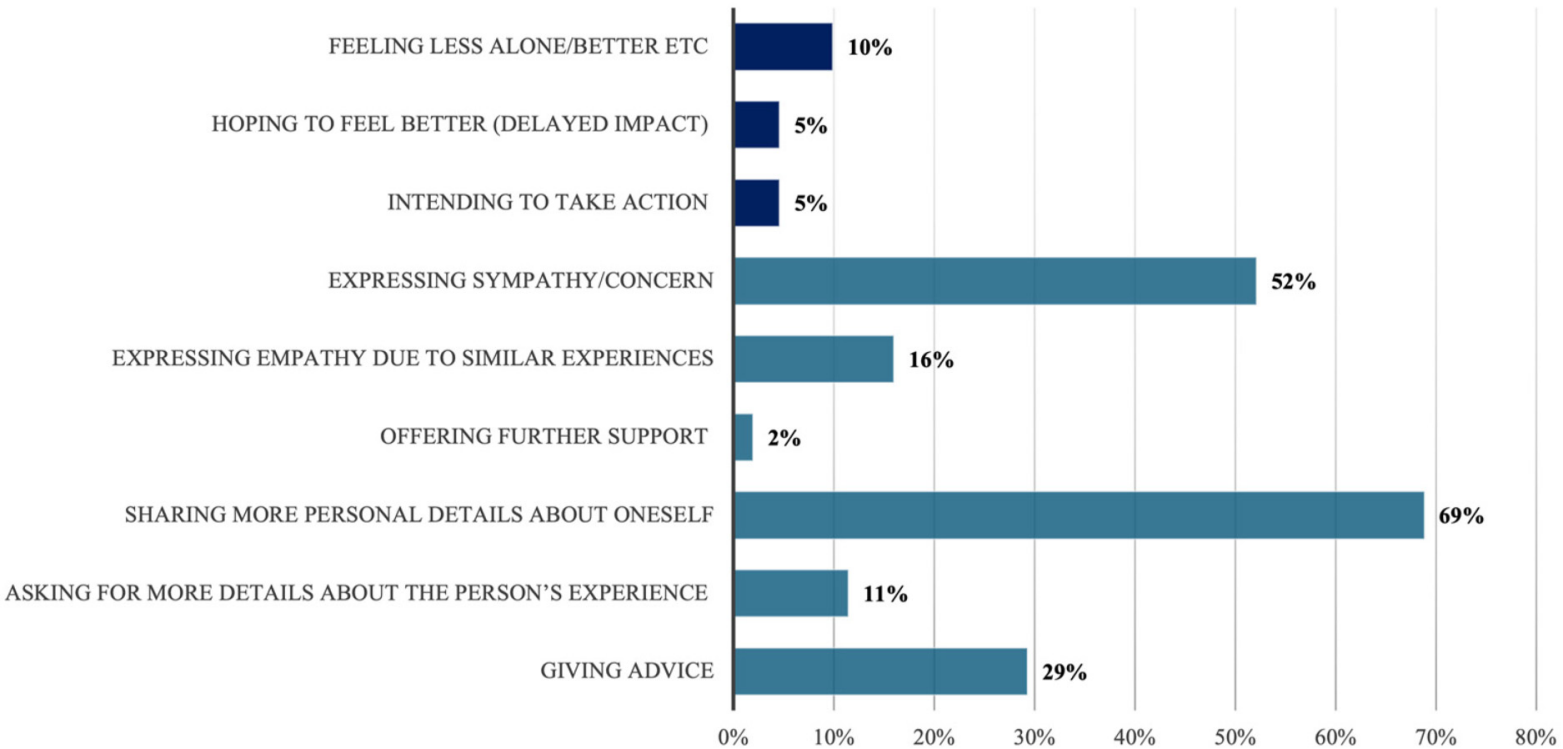
Distribution of the impact and interactional codes across posts that include *sharing your experience* (% of posts in which each code is present).

A breakdown of the above information by individual codes is provided in Figure 3, with the three codes we grouped under ‘impact’ at the top and the remaining interactional codes below.

The ‘expressing sympathy/concern’ code (example 12) and the ‘expressing empathy due to similar experiences’ code (example 13) occur, respectively, in 52% and 16% of posts that include *sharing your experience*. They apply to posts that tend to present the addressee as being in a difficult position, and the poster as relating to them emotionally:

(12) Thanks for **sharing your experience** with us in such an open and honest way. It seems like you have had a difficult time …
(13) Thank you for **sharing your experience**, it is a help when I read that I’m not the only one who feels like this.

The interactional code ‘sharing more personal details about oneself’, which occurs in 69% of posts, typically captures cases where the poster discloses difficult experiences from their own lives as a result of reading similar experiences shared by someone else, as in example 14 (NB: This example was also coded for ‘expressing empathy due to similar experiences’):

(14) Thank you for **sharing** some of **your experiences** - […] It sounds so hard to grow up with a [CONDITION]. I also have a history of [CONDITION], and my family didn't speak about it either …

The code ‘asking for more details’, which occurs in 11% of posts, engages the addressee in further dialogue, as in example (15):

(15) I’m really considering again getting some private therapy in the meantime. Do you mind if I ask whether there's anything that you did differently, that you would say helped you to avoid […]? Thank you, really appreciate you **sharing your experience**, it does make me feel better

Among the ‘impact’ codes the most frequent is ‘feeling less alone/better/supported’, which occurs in 10% of posts. This applies to example (15) above, where the poster explicitly says: *it does make me feel better.* In the 5% of posts that received the code ‘hoping to feel better’, the improvement in the person's and/or family member's situation as a result of reading someone else's experience is anticipated, rather than already realised, as in example (16):

(16) I am so looking forward to the day my wife feels that joy and peace again. […] Everything that's happened has only made me love her more […] Thank you **for sharing your** own **experiences** of courage and strength with me, I’ll always be grateful to you

The code ‘intending to take action’, which occurs in 5% of posts, typically applies to cases where the poster states that, as a result of reading someone else's experience, they will seek help or resume medication, as in *I will start taking [MEDICATION] again* in example 17 (NB: Example (17) was also coded as ‘feeling less alone/better/supported’):

(17) Thanks so much for **sharing your experience**, it has really put my mind at ease. And you’re right, people get sick and that's just part of life. I see lined [lived] experience of struggles with mental health as something positive in healthcare. I think I will start taking [MEDICATION] again.

As example (16) shows, such reactions may also occur in posts by family members, who equally express gratitude to the posters who shared their experiences. Example (17) additionally highlights the value of the pool of shared experience, as in: *I see lined [lived] experience of struggles with mental health as something positive in healthcare*.

## Discussion

Earlier studies,^9–15^ using different methodologies and approaches, show that participating in online communities, seeking emotional support, information and peer network, may potentially lead to improved self-management and enhanced subjective psychological well-being, and better prepare patients for consultation with medical professionals. The findings of our study further support, contextualise and expand these earlier findings.

Our analysis of the use of *in/from my experience* (RQ1) shows that, when people explicitly refer to their personal *experience*, they predominantly use it to provide support for others via information and/or advice, with information appearing in the largest proportion of posts (87%). The information/advice may concern the mental health condition itself, how to manage the condition (through medication or non-clinical approaches) and how to navigate healthcare systems (by accessing them in the first place and then dealing with different kinds of health professionals). Over half of posts (57%) contained information on condition, treatment and medication. This highlights some of the potential positive functions of online forums, that is, to provide a space in which lived experience can be explicitly used with an altruistic intent and potentially beneficial effects.

In addition to lay advice and information exchange, the online conversations have been found to often be affectionate and emotionally supportive, which may be in contrast to time limited encounters with busy healthcare professionals.^51^ Our quantitative and qualitative findings confirm that, when posters employ the phrases *in/from my experience* on our two forums, they have, at least in part, an addressee-oriented focus, that is, they use events that occurred in their own lives to support and contextualise the provision of advice or information that is intended to benefit a specific forum contributor or members of the online community more generally. Similarly, when experience of others is received, referred to by posters as *your experience*, it shows significant levels (91% of posts) of interactional orientation, for example, expressing sympathy, empathy, offering support (example 19), giving advice (example 18). This helps to construct the online communities as safe, supportive spaces with strong social bonds among contributors.

(18) Hi X Thanks for **sharing your experience** […] I think, as X suggested, that if you consult with Prof X that will probably help you to understand your mood swings and feelings of anxiety better.
(19) Thanks for **sharing your experiences** […] it appears that you have put some good plans in place. Do please come back if you have more questions to share […], take care, xx

Moreover, while we found instances of, for example, blanket negative judgements about health professionals, we did not find any clearcut instances of what we would describe as mis/disinformation or potentially harmful advice. It is not possible from the data available to know whether such posts are not being produced, or have been edited out as part of the moderation process. Other studies of peer online interactions have identified levels of information inaccuracy or information not fitting the needs of the patients.^29,52,53^

Our linguistic approach has also shown that, while drawing from one's experience to dispense advice and/or helpful information involves claiming some degree of authority for oneself, some posters use explicit disclaimers to indicate that their own experience may not be applicable to everyone else and/or cannot substitute professional opinion. In both examples (20) and (21), for example, the posters emphasise that they are speaking *only* from their experience, while in example (21), the poster additionally suggests that they may be *very wrong.* Some kind of disclaimer was identified in 8.4% of the occurrences.

(20) I can't speak from the point of view of a doctor so can **only** talk **from my experience**…
(21) Unfortunately, it's not possible for me to comment on the particularities of your case as I don't know. The information that I can share is **only from my** own **experience** and **it's possible that I am very wrong**.

This suggests, in line with findings from other studies,^54^ that online communities may develop self-regulated practices that improve the quality of the information peer exchange.

With regard to the use of *sharing your experience* (RQ2), the presence of thanks or some other form of appreciation alongside the phrase confirms the extent to which forum contributors value reading other people's experiences. While we have not further explored the role of gratitude expressions, the high frequency of these expressions suggests its non-peripheral role in the online discourse.^55^ We also found some evidence of potential subjective positive impacts of receiving others’ experiences, both on how people feel and on what they plan to do about their mental health issues. In most cases, however, posts containing the phrase *sharing your experience* provide evidence of how experience-sharing contributes to mutual support, social bonding and further engagement on the forum. We showed how forum contributors expressed sympathy or empathy with the person who had shared the experience, disclosed further details about themselves, or invited further interaction by asking questions or giving advice. This suggests a virtuous cycle in which sharing lived experience facilitates others to also feel able to share and get support from the forum.

As with *in/from my experience*, we cannot know if moderators deleted posts containing *sharing your experience* that might challenge the addressee or talk about negative impacts, but we did not find evidence of this in our analysis. Rather, we found clear evidence that the sharing of experience is appreciated and that it is conducive to supportive relationships on the forum, and potentially positive impacts outside the forum.

By introducing a complementary methodology for the study of spontaneous online conversations, we have also shown the value of interdisciplinary approaches. Through the construction of a highly relevant, clearly delimited, dataset, based on one salient keyword (*experience*), we were able to analytically move between quantitative and qualitative findings. We developed a bottom-up coding scheme that reflects the nature and content of our dataset. Both the coding scheme and the findings of our study confirm some of the findings in earlier studies while offering a more nuanced additional perspective via a detailed snapshot of linguistic behaviour in two online communities.

Our study was, however, limited both in scope and sample size. In addition, the anonymisation of the data did not make it possible to distinguish systematically between posters and moderators, and posters with different degrees of involvement in the online community. Some earlier studies suggest that some online communities tend to have core groups of a limited number of users that provide most of the information and advice to more peripheral participants.^56^ Some of the specialised sub-forums in Magpie in our data suggest similar behaviour that invites further investigation. Similarly, earlier studies show that the process of sharing information and advice online varies across different social networks, with some of the online platforms being more informative and others containing more messages of social and emotional support.^10,57^ These differences may be due to the nature of the health condition, the gender of the contributors,^58^ or the underlying ethos of the community.^10^ While this was not the focus of our study, we have come across similar patterns of behaviour, pointing to further potential areas of research interest.

While our results highlight the importance of the forums in creating a safe space for sharing information and advice about managing mental health challenges and navigating health services, based on lived experience, they also suggest that forums are perceived primarily as supplements to traditional care, especially vis a vis long waiting times for face-to-face care, rather than as an alternative.

## Conclusion

Peer online forums dedicated to mental health are increasingly being described and used alongside or as an alternative to mental health services, in the United Kingdom and around the world. Such forums are often described as valuable because they provide opportunities for lived *experience* to be shared, potentially for the benefit of both those who disclose it and those who receive it. However, research findings are mixed with regard to positive versus negative impacts of mental health online forums.

Our analysis of real-world forum posts offers a way to see directly how sharing of lived experience works in online forums without being filtered through the reflections of the participants, or the social desirability and demand characteristics of surveys or interviews. Great care was taken to do this within an ethical framework that ensured users would remain anonymous. However, the analysis was limited to posts from only two of the seven forums collaborating on the iPOF project, and only on a selection of explicit references to *experience*. In this particular study, we did not identify any notable differences between these two forums and the analysis results are not presented comparatively. However, as discussed above, the analysis pointed to directions of potential future research that may reveal patterns of behaviour that are specific to some of the forums and may not be generalisable. Therefore, care is needed in generalising the results to other forums with different design, different user demographics, different topics and moderation features. We have also considered posts individually, rather than as part of longer interactions, and we have not included any information that may be available about the forum contributors and their circumstances.

Peer online forums offer a free, widely accessible space for people to access support through the sharing of lived experience by people who are facing similar health challenges. Whilst highlighting some of the difficulties in accessing routine offline mental health services, the information and advice are generally in favour of engagement with services, suggesting that forums could offer a gateway into services, or a useful adjunct to other forms of support, and potentially help to alleviate distress for those on long waiting lists. Further research is needed to test the generalisability of these findings to other forums for mental health, and for other health conditions. More work is also needed to understand the range of impacts of using peer online forums on users, and their use of other services. This could inform policy and commissioning decisions around the role online forums should play in health service delivery.

### Lived experience commentary by J. S. and Neil Caton

The use of lived experience within mental health forums has the potential to play a key role in a range of different outcomes for both forum contributors and forum readers. As a moderator, aiming to ensure that forum contributors speak from their own lived experience has been crucial. It creates a space for a broader range of experiences to be shared, accepted, and interacted with which has helped in facilitating many of the positive impacts which have been highlighted in this research.

However, it has also been highlighted that the available data limits the ability of knowing whether some instances of negative blanket statements or misinformation are not being made or have been amended by moderators. Given that the forums chosen had active moderation, how might this compare with forums that did not? This is an area which could be important to explore further, particularly in relation to the potential impacts of different moderating styles and/or rules on forum contributors, readers, and perhaps on moderators themselves. It is also worth exploring whether and to what degree forum contributors might feel able to speak from their own lived experience on different forums and under different styles of moderation. Factors such as certain topics or keywords being banned or the potential for bias when applying forum rules could lead to a range of impacts on sharing lived experience.

Overall, it is reassuring that this research highlights how valuable it can be to share lived experiences within mental health forums. Further research in this area has the potential to lead to positive changes in how moderators accommodate lived experience. It also has the potential to lead to more collaboration with external service providers. It will be interesting to see how further research evolves and influences change in ways which provide hope whilst remaining cautious of ongoing risks and suitability.
